# Temperature-tunable lasing from dye-doped chiral microdroplets encapsulated in a thin polymeric film

**DOI:** 10.3762/bjnano.9.37

**Published:** 2018-01-31

**Authors:** Gia Petriashvili, Mauro Daniel Luigi Bruno, Maria Penelope De Santo, Riccardo Barberi

**Affiliations:** 1 Institute of Cybernetics of the Georgian Technical University, Tbilisi 0175, Georgia; 2 Physics Department, University of Calabria, Rende 87036, Italy; 3 CNR-Nanotec UOS di Cosenza , c/o University of Calabria, Rende 87036, Italy

**Keywords:** chiral microdroplets, dye-doped cholesteric liquid crystals, laser, polymeric films, temperature tuning

## Abstract

In the last decade, much interest has grown around the possibility to use liquid-crystal droplets as optical microcavities and lasers. In particular, 3D laser emission from dye-doped cholesteric liquid crystals confined inside microdroplets paves the way for many applications in the field of sensors or tunable photonics. Several techniques can be used to obtain small microresonators as, for example, dispersing a liquid crystal inside an immiscible isotropic fluid to create an emulsion. Recently, the possibility to obtain a thin free-standing film starting from an emulsion having a mixture of water and polyvinyl alcohol as isotropic matrix has been reported. After the water evaporation, a polymeric film in which the microdroplets are encapsulated has been obtained. Bragg-type laser emission has been recorded from the emulsion as well as from the thin film. Here, we report on the possibility to tune the laser emission as a function of temperature. Using a chiral dopant with temperature dependent solubility, the emitted laser wavelength can be tuned in a range of 40 nm by a temperature variation of 18 °C. The proposed device can have applications in the field of sensors and for the development of anti-counterfeiting labels.

## Introduction

Liquid crystalline materials show peculiar optical properties. Cholesteric liquid crystals (CLCs), in particular, can be regarded as an example of self-assembled one-dimensional photonic crystals. In fact, molecules in CLCs self-assemble in a chiral supramolecular structure. The helical structure leads to a periodic variation of the refractive index inside the material. Due to the inversion symmetry of the molecules, the periodicity of the structure is half the helix pitch, *p*. When light propagates along the helical axes, it will experience Bragg reflection at λ_0_ = *n*p, where λ_0_ is the wavelength of the maximum reflection and *n* is the average of the refractive indices defined as: *n* = (*n*_e_ + *n*_o_)/2, where *n*_e_ and *n*_o_ are the extraordinary and ordinary refraction indices, respectively. As a consequence, a whole range of wavelengths does not propagate inside the material and it is indicated as photonic band gap (PBG). The full width at half maximum of the PBG equals to Δλ = *p*Δ*n*, where Δ*n* = *n*_e_ − *n*_o_ is the birefringence of the liquid crystal. The PBG spectral position is sensitive to external or internal factors as electric and electromagnetic fields, temperature and local order variations [1–4]. Due to the presence of a PBG, CLCs behave as Bragg resonators that can be used to build up mirror-less lasers. In fact, when a CLC is doped with a fluorescent dye, the latter plays the role of the active medium inside a resonator. In 1980, for the first time Ilchishin demonstrated lasing from a dye-doped CLC (DD-CLC) [5]. Since then, laser emission has been demonstrated in several CLC based systems [6–8]. Further, since the PBG spectral position can be shifted, also the laser emission wavelength can be tuned by using external factors as the temperature variations. CLCs are often mixtures of nematic liquid crystal and chiral dopant. A chiral dopant with temperature dependent solubility allows for a shift of the PBG position [9–10]. Increasing the temperature, more chiral molecules become dispersed in the mixture winding the cholesteric helix and leading to a shift of the photonic band toward shorter wavelengths. By choosing a proper dye, a shift of the emitted laser wavelength is observed [11–12].

In recent years, several studies have been published on the CLCs encapsulation in micrometer-sized spherical objects [13]. Inside the microdroplets the CLCs helical structure is preserved and, for boundary planar conditions, the helices axes are radially oriented. When CLCs are doped with fluorescent dyes, 3D omnidirectional laser emission from microdroplets is observed [14–15]. This effect is extremely interesting for those who work in manufacturing micro-optical components and integrated optical circuits. Several techniques are used to prepare CLCs microdroplets for Bragg laser emission [16–17] and, also in this case, the emitted laser wavelength can be tuned varying the temperature. In [18], for example, Humar reported a wavelength tuning of about 50 nm for a temperature variation of 14 °C.

Recently, we have proposed a method to prepare emulsions containing different kinds of cholesteric liquid-crystal microdroplets in order to obtain simultaneous Bragg-type laser emission at different wavelengths [17]. Following a drying procedure these emulsions can be transformed into free-standing polymeric films in which the lasing characteristics are preserved. In this work, we demonstrate that the laser emission tuning can be obtained preparing the CLC mixture by using a chiral dopant with temperature-dependent solubility. For both emulsion and thin film form, the laser emission wavelength can be tuned. In particular, laser emission from the thin film is tuned over a range of about 40 nm for a temperature variation of 18 °C. The tuning proves that, even if the droplets are embedded in a polymeric matrix, the liquid crystalline structure is fluid enough to allow the dissolution of more chiral dopant inside the nematic liquid crystal, increasing the winding of the cholesteric helix.

## Results and Discussion

The materials used for the emulsion preparation are described in the following. The nematic material ZLI-1939, with a birefringence of 0.18, and the chiral dopant ZLI-811, both from Merck, are used. The cholesteric liquid crystal mixture is prepared with the following percentages: 70.5 wt % ZLI-1939 + 29.5 wt % ZLI-811. The chiral dopant is chosen for its well-known temperature-dependent solubility [9]. The chiral mixture is confined inside a 36 μm cell the internal surfaces of which are covered with a rubbed thin layer of polyvinyl alcohol (PVA). The PVA (Sigma-Aldrich) is deposited by spin-coating a solution containing 0.5 wt % of the polymer in water. An optical fiber, coupled to a spectrometer (AVASPEC-2048, Avantes) with 1 nm resolution, is used to collect the light emitted from the sample.

**Figure 1:**
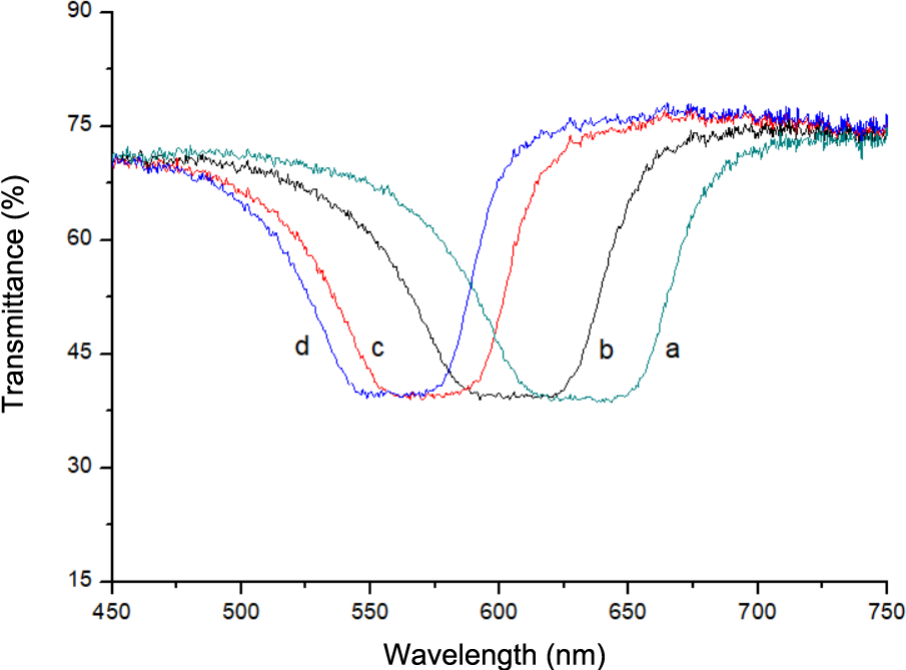
Spectral shift of the PBG as a function of the temperature: a) 12 °C (cyan line), b) 17 °C (black line), c) 22 °C (red line) and d) 25 °C (blue line).

To detect the transmission spectra as a function of temperature, the cell is confined inside a heater (CaLCTec, mod. FB-250). Figure 1 shows that temperature increase leads to a shift of the PBG towards shorter wavelengths. The cyan line was acquired at 12 °C, the black line at 17 °C, the red line at 22 °C and the blue line at 25 °C. When temperature increases, more chiral dopant goes in solution and the pitch of the cholesteric helix shortens.

**Figure 2:**
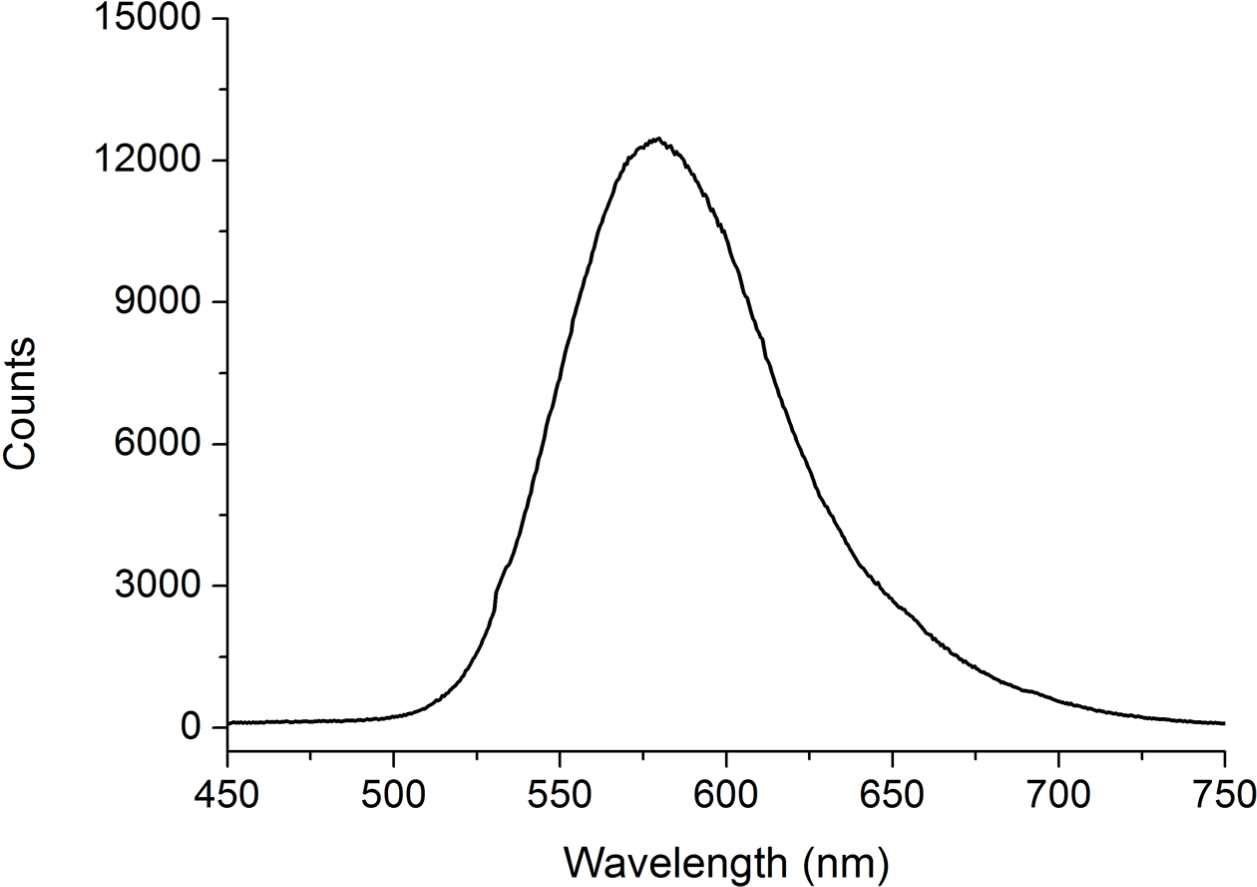
DCM fluorescence spectrum.

For lasing experiments, the dye fluorescence spectrum must overlap the whole shifting interval of the PBG. The selected fluorescent dye is 2-[2-[4-(dimethylamino)phenyl]ethenyl]-6-methyl-4*H*-pyran-4-ylidene]propanedinitrile (DCM) from Exciton. It is added to the chiral mixture with a concentration of 0.3 wt %. Figure 2 shows the fluorescence spectrum of the DCM dye.

First, the dye-doped CLC mixture is used to prepare the emulsion. With respect to [17], the emulsion is prepared with the same procedure, but it contains only one type of dye-doped CLC prepared using different compounds. A small amount (ca. 1 wt %) of the DD-CLC is deposed in a glass bottle and a solution of PVA, 10% (w/w) in water, is added to it. The solution is mixed at a temperature of 90 °C for at least 30 min. The bottle is placed on a laboratory vortex mixer and shaked for 30 s in order to produce the emulsion. Since water and liquid crystal are immiscible, this procedure is functional to the formation of DD-CLC microdroplets. The obtained droplet sizes range between 10 and 60 μm. As reported in [17], PVA plays the double role to stabilize the emulsion avoiding the coalescence of droplets and to induce the planar alignment of the liquid-crystal molecules at the interface. Inside the droplets, the cholesteric helices arrange in a radial configuration, with the cholesteric layers bent in concentric spherical surfaces.

**Figure 3:**
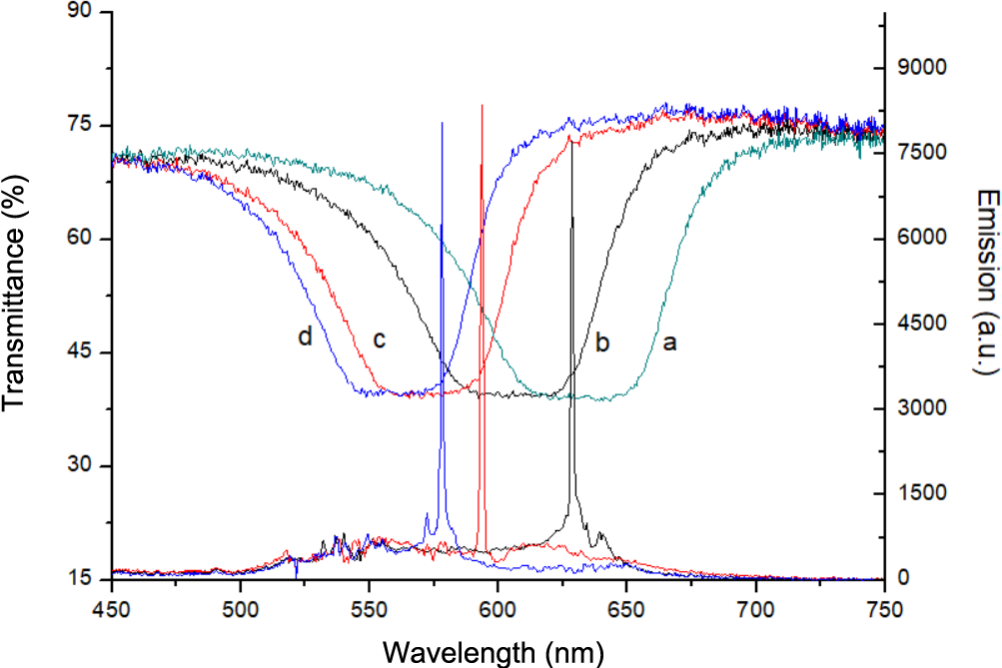
Blue shift of the emitted laser wavelength.

In order to study laser emission from these microstructures, the emulsion is confined in a cuvette and pumped with the second harmonic of a Q-switched Nd:YAG laser (Continuum Surelite II). The pulse wavelength, width and repetition rate are 532 nm, 4 ns and 5 Hz, respectively. The pulse energy density is 6 mJ/cm^2^. The cuvette is placed on a heater and the temperature is left to stabilize. The Bragg-type laser wavelength is recorded using an optical fiber placed off-axis with respect to the pumping beam. Since the lasing properties of the emulsion are not investigated at the level of the single microdroplets, a 30% variation of the laser peak intensity is observed while its spectral position slightly changes by about ±2 nm. Figure 3 shows the lasing tuning measured by increasing the temperature. Laser emission is recorded at 578 nm, 594 nm and 628 nm, corresponding to a wavelength tuning range of about 50 nm. As previously observed, the temperature increase causes a shift of the laser emission towards shorter wavelengths. Since the cuvette is heated from below while the pumping beam is focused in its middle, it is very difficult to determine the droplets temperature at that position. In [11] the authors report that, depending on the temperature and on the spectral position of the dye fluorescence with respect to that of PBG, laser emission can be observed at the right and/or left edge of the PBG. Then, it is impossible to provide an estimate of the droplets temperature using the spectral position of the laser wavelength. Nevertheless, we could assume that at low temperatures, laser emission is more likely to occur at the left edge of the PBG that is closer to the maximum of the fluorescence with respect to the right edge, while at higher temperatures the emission is more likely to occur at the right edge of the PBG. Since the observed shift is larger than the width of the PBG, we can assert that the laser tuning from 628 nm to 578 nm is an effect due to the temperature increase.

**Figure 4:**
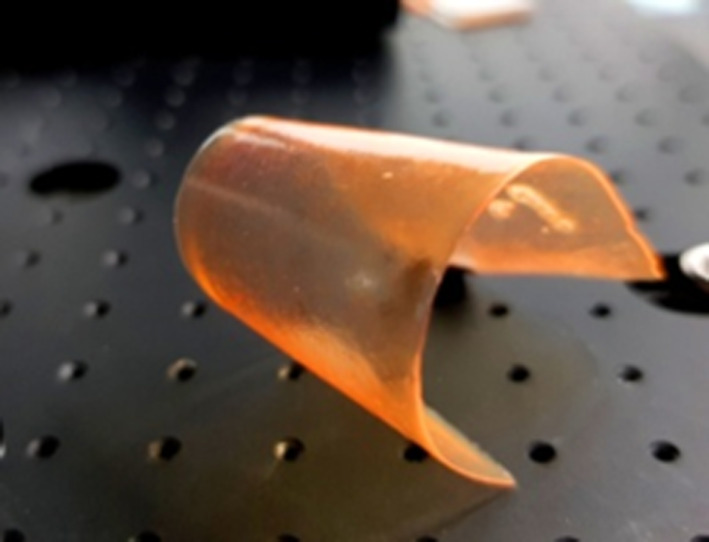
Polymeric film containing the DD-CLC microdroplets obtained after water evaporation.

In a further step, the emulsion is drop-cast on a laboratory glass slide and left to evaporate for 48 h at room temperature as described in [17]. After water evaporation, the formed thin film can be easily detached from the glass (Figure 4).

**Figure 5:**
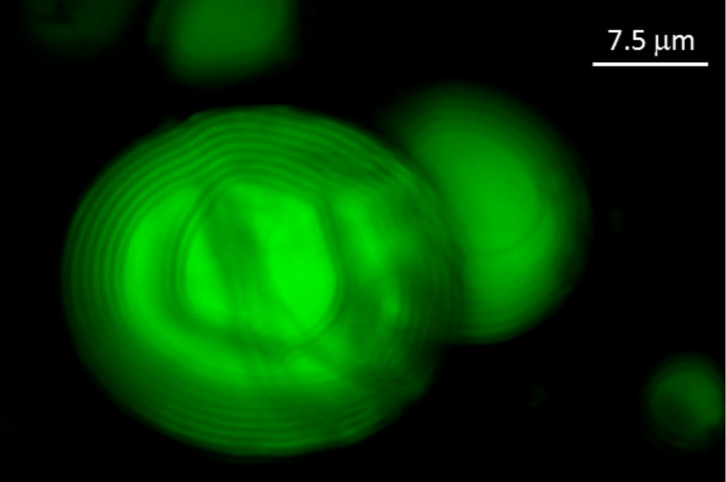
Confocal micrograph of two DD-CLC microdroplets confined in a polymeric film.

In Figure 5, a picture of the microdroplets obtained through confocal microscopy (Leica,DM6000 TCS SP8) is shown, which confirms the stabilization through PVA by preventing the coalescence of droplets during the drying process. The periodicity of the CLC droplets, shown in the picture, was about 1.36 μm. The pitch was purposely selected to overcome the resolution limits of the used confocal microscope. Although the microdroplets are deformed during the drying procedure, their internal periodicity is clearly visible.

**Figure 6:**
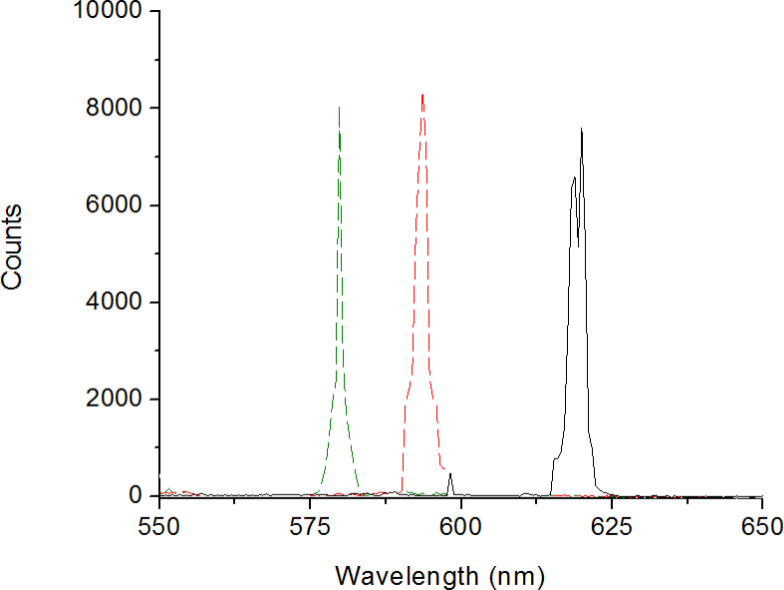
Blue shift of the emitted laser wavelength, measurements acquired at 28 °C (black solid line), at 40 °C (red dashed line) and at 46 °C (green dashed line), respectively.

Laser emission as a function of temperature is studied confining the film inside the heating stage. The thin film is optically pumped using the second harmonic of a Nd:YAG laser and the emitted laser spectrum is recorded (Figure 6). The optical fiber that collects the laser emission is placed off-axis with respect to the impinging beam.

The first lasing peak is observed at 620 nm for 28 °C (Figure 6, black solid line), a higher temperature than the one used for the emulsion. Interestingly, increasing the temperature, it is still possible to increase the solubility of the chiral dopant molecules inside the liquid crystal microdroplets and to obtain a consequent winding of the helix. In fact, the emitted laser peak shifts to 594 nm for 40 °C (red dashed line) and to 580 nm for 46 °C (green dashed line). In a temperature interval of 18 °C, the emitted laser wavelength is tuned over a range of 40 nm. The temperature values needed to record a shift of the emitted laser wavelength are larger compared to the ones needed for the simple emulsion. This phenomenon could be ascribed to the presence of PVA molecules inside the droplets, which cause a mobility reduction of the chiral dopant molecules. As a consequence, higher temperatures are needed to solubilize the chiral dopant.

## Conclusion

Temperature-tunable lasing from self-organized helical DD-CLC microdroplets trapped in a polymeric film is obtained. The tuning was observed over a range of 40 nm, achieving about the same range as the one observed in a PVA/water emulsion. Films are easy to prepare, self-supporting and flexible. The presented device is promising for future microphotonics and anti-counterfeiting applications.
